# Atomically Dispersed High-Active Site Density Copper Electrocatalyst for the Reduction of Oxygen

**DOI:** 10.3390/ma17205030

**Published:** 2024-10-15

**Authors:** Tao Jiang, Hongli Jiang, Weibin Wang, Hao Mu, Ying Zhang, Bo Li

**Affiliations:** 1 Electric Power Research Institute of Guizhou Power Grid Co., Ltd., Guiyang 550002, China; muhao0610@sina.com (H.M.); zhangyingmails@126.com (Y.Z.); gzgylb2207@163.com (B.L.); 2 School of Intelligent Manufacturing, Zhejiang Dongfang Polytechnic, Wenzhou 325000, China; 17816038523@163.com

**Keywords:** oxygen reduction, catalyst, Cu–N_4_, active site density

## Abstract

Enlarging the M-Nx active-site density is an effective route to enhance the ORR performance of M-N-C catalysts. In this work, a single-atom catalyst Cu–N@Cu–N–C with enlarged Cu–N_4_ active site density was prepared by the second doping and pyrolysis (SDP) of Cu–N–C derived from Cu-doped zeolite imidazole frameworks. The half-wave potentials of Cu–N@Cu–N–C were measured as 0.85 V in alkaline electrolyte and 0.75 V in acidic media, which was 50 mV and 60 mV higher than that of Cu–N–C, respectively. N_2_ adsorption–desorption isotherm curves and corresponding pore distribution analysis were used to verify the successful filling of additional Cu and N in micropores of Cu–N–C after SDP. The obvious increase in Cu contents for Cu–N@Cu–N–C (1.92 wt%) compared with Cu–N–C (0.88 wt%) tested by ICP demonstrated the successful doping of Cu into Cu–N–C. XAFS analysis confirmed the presence of Cu–N_4_ single-atom active centers in Cu–N@Cu–N–C. The N 1 s high-resolution XPS results proved a great increase in Cu–N_4_ contents from 13.15% for Cu–N–C to 18.36% for Cu–N@Cu–N–C. The enhanced ORR performance of Cu–N@Cu–N–C was attributed to the enlargement of Cu–N_4_ active site density, providing an effective route for the preparation of efficient and low-cost ORR catalysts.

## 1. Introduction

The development of platinum group metal (PGM)-free oxygen reduction reaction (ORR) catalysts with high performance and low cost is still significant for the application of devices like fuel cells and metal–air batteries [1,2,3,4]. Transition metal and nitrogen co-doped carbon (M–N–C, M=Fe, Co, Mn, Cr, Ni, et al.) species are the most reported PGM-free ORR catalysts that have outstanding catalytic performance comparable to commercial platinum (Pt) catalysts [5,6,7,8,9,10]. M–N_x_ species such as Fe–N_4_, Co–N_4_, Mn–N_4_, and Cr–N_4_ are commonly demonstrated as active centers for M–N–Cs in ORR [11,12,13,14,15,16]. However, metallic, oxidized, and even agglomerated species are generally formed accompanied by the formation of M–Nx during the calcination process [17,18,19]. These species are generally not sufficient for ORR, and we should minimize their presence. Single-atom catalysts (SACs) with atomic-level distribution of metal elements can solve this problem very well [12,20]. SACs can ensure the most efficient utilization of metal atoms of M-N-Cs and fully expose their M–Nx active centers [21,22]. Metallic oxides have been demonstrated with notable ORR catalytic performance. For instance, spherical TiO_2_ nanoparticles with a size of 18 nm are reported to act as a good alternative for Pt-free ORR catalysts [23]. However, TiO_2_ usually catalyzes ORR with a two-electron pathway [24]. Semiconductor materials like Cu_2_O and Fe_2_O_3_ are also reported to show good ORR performance [25,26,27]. Cu_2_O and N co-doped nanocarbon materials are used as activated cathode catalysts for microbial fuel cells [28]. Fe_2_O_3_ nanoparticles have good ORR activity and are usually modified with 1D or 2D N-doped carbon to serve as efficient catalysts [29,30]. Metal-free ORR catalysts like graphene, carbon nano tubes, nanocarbon matrixes, and N-doped carbon materials (especially Pyridinic-N and Graphitic-N) can catalyze ORR, but most of them prefer inefficient two-electron pathways and inferior long-term stability [31,32,33,34,35,36]. In general, metallic oxides and metal-free species have much lower catalytic activity compared with M-N-Cs, according to most published works. It should be noted that M–N–Cs display relatively poor performance when assembled in devices like fuel cells or Zn–air batteries compared with precious metal-based catalysts. Furthermore, most M-N-Cs demonstrate inferior ORR performance in acidic environments, which can be considered as a potential application for proton exchange membrane fuel cells. Fe–N–C species have the most outstanding ORR performance among M–N–Cs. However, they always suffer from the Fenton effect, which hinders their ORR activity and stability because of the possible solubility of Fe-Nx in the presence of H_2_O_2_ [37,38,39,40]. Co–N–C species show superior ORR performance that is comparable to Fe–N–C. But they usually suffer from poor dispersion of Co elements [41,42,43,44]. Moreover, Mn–N–C and Cr–N–C have also been reported to perform good ORR catalytic activity in alkaline or acidic electrolytes and even in proton exchange membrane fuel cells [6,45]. Cu–Nx coordination has been reported to catalyze ORR efficiently with a lower overpotential (only 20 mV, lower than most of Fe-Nx, Co–Nx, and even Pt/C) [46]. However, it is noteworthy that Cu catalysts with efficient ORR performance are rarely reported.

Excellent ORR activity generally requires high specific surface area (SSA), high electron conductivity, and high active site density [47,48,49]. Metal–organic frameworks (MOFs) with superior electron conductivity, large SSA, and hierarchical pores that are easily doped are widely used as precursors to prepare supercapacitors, photocatalysts, molecular sieves, and electrocatalysts [50,51,52,53]. Zeolite imidazole framework–8 (ZIF–8) with Zn^2+^ as metal centers and imidazole as ligands is the most common precursor in the preparation and design of ORR catalysts [41,54,55,56]. By directly pyrolyzing ZIF–8, nanocarbon materials with large SSA and hierarchical porous structures can be obtained [54,57,58]. By replacing Zn^2+^ with transition metal ions like Fe^3+^ (or Mn^2+^, Cr^3+^, et al.), serving as metal centers, Fe can be adsorbed into 3D nanostructures during the growth process of ZIF–8 [1,6,8,12,42,59]. 

The shortcoming of PGM-free catalysts still lies in their insufficient activity, which can be solved by increasing active site density and achieving the atomic level distribution of transition metal elements [60,61]. The active site density of M–N–C species can be enlarged by filling their micropores with M–Nx. Researchers reported the additional formation of Mn-N_4_ in the micropores of Mn-N-C to enhance its acidic ORR performance [6]. Herein, we introduce more Cu–N_4_ active centers into the micropores of Cu–N–C to enrich its active site density and prepare a Cu–N@Cu–N–C catalyst using a second doping and pyrolysis method. A great increase in Cu–N_4_ contents is obtained after the addition of Cu and N into the micropores of Cu–N–C. XAFS analysis verifies the atomic level distribution of Cu in Cu–N@Cu–N–C catalyst and confirms the Cu–N_4_ active centers. Electrochemical measurement results in alkaline media indicate the enhanced ORR activity from E_half_ = 0.80 V to E_half_ = 0.85 V after the enlargement in Cu–N_4_ active site density. In acidic electrolytes, the ORR performance also enhances from E_half_ = 0.69 V to E_half_ = 0.75 V. The enhancement in ORR activity is attributed to the enlarged single-atom Cu–N_4_ active site density, which accelerates the transmission of electrons and speeds up the breaking of O=O bonding.

## 2. Materials and Methods

### 2.1. Preparation of Cu–N–C

The synthesis of Cu–ZIF–8 was similar to our previous work. Firstly, zinc nitrate hexahydrate (Zn(NO_3_)_2_·6H_2_O) and copper acetate monohydrate Cu(CO_2_CH_3_)_2_·H_2_O (molar ratio: Cu/Cu+Zn = 20%) were dissolved in methanol, followed by the addition of methanol containing dimethyl imidazole. Secondly, the above mixed solution was stirred for 24 h at room temperature. Then, the above solution was collected after centrifugal cleaning with methanol three times. Finally, Cu–ZIF–8 was collected after being dried at 60 °C for 12 h in a vacuum oven. To prepare the Cu–N–C sample, firstly, Cu–ZIF–8 was directly pyrolyzed at 900 °C and kept for 2 h in an Ar atmosphere with a heating speed of 5 °C min^–1^. Secondly, the above collected black powders were etched in 0.5 M H_2_SO_4_ at 80 °C for 5 h, followed by washing with water and ethanol at least four times. Finally, Cu–N–C was collected after vacuum drying at 60 °C for more than 12 h. 

### 2.2. Preparation of Cu–N@Cu–N–C

Firstly, 150 mg of Cu–N–C were dispersed into a mixed solution containing 50 mL isopropanol and 50 mL water. Secondly, 12 mg (0.07 mmol) of copper (II) chloride dihydrate (CuCl_2_·2H_2_O) and 75 mg (0.60 mmol) of melamine were added to the above solution. Thirdly, the mixed solution was ultrasonicated for 2 h and stirred for 6 h at room temperature, followed by washing with ethanol two times and drying in a vacuum oven. Then, the collected dried powders were pyrolyzed at 900 °C for 2 h in an Ar atmosphere with a heating speed of 5 °C min^−1^. Finally, Cu–N@Cu–N–C was successfully prepared after acid etching in 0.5 M H_2_SO_4_ at 80 °C for 5 h, followed by washing with water and ethanol at least four times and vacuum drying.

## 3. Results and Discussion

### 3.1. Synthesis and Microscopic Characterization of Catalysts

**Figure 1:**
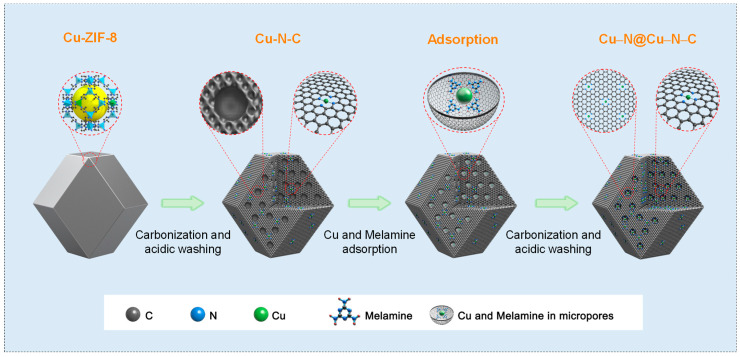
Schematic illustration showing the preparation of Cu–N–C and Cu–N@Cu–N–C catalysts.

The synthesis of Cu–N–C and Cu–N@Cu–N–C catalysts is schematically presented in Figure 1. Cu–ZIF–8 was synthesized when Cu ions partially replaced Zn ions during the traditional preparation process of ZIF–8 [62]. The direct carbonization of Cu–ZIF–8 in the Ar atmosphere followed by acidic etching in 0.5 M H_2_SO_4_ solution was used to prepare the original Cu–N–C catalyst. It is apparent that a large number of Cu–Nx active sites exist on the surface of Cu–N–C carbon nanomaterials, similar to that of typical Fe–N–C, Cr–N–C, and Co–N–C species. Moreover, abundant, evenly distributed micropores around the three-dimensional structures of Cu–N–C can be observed.

In this work, additional Cu–Nx active sites were predicted to anchor into the micropores of Cu–N–C, attempting to increase its active site density and enhance its ORR catalytic performance. The typical approach was as follows. Firstly, additional Cu salts and organic ligands were adsorbed into the micropores of Cu–N–C by the simple solvent method. Secondly, a second pyrolysis in an inert atmosphere was used for the carbonization of organic ligands and the formation of additional Cu–Nx. Thirdly and finally, the carbonized products were etched in 0.5 M H_2_SO_4_ solution to remove the possible metallic clusters, and the Cu–N@Cu–N–C catalyst with high Cu–Nx active site density was successfully prepared.

**Figure 2:**
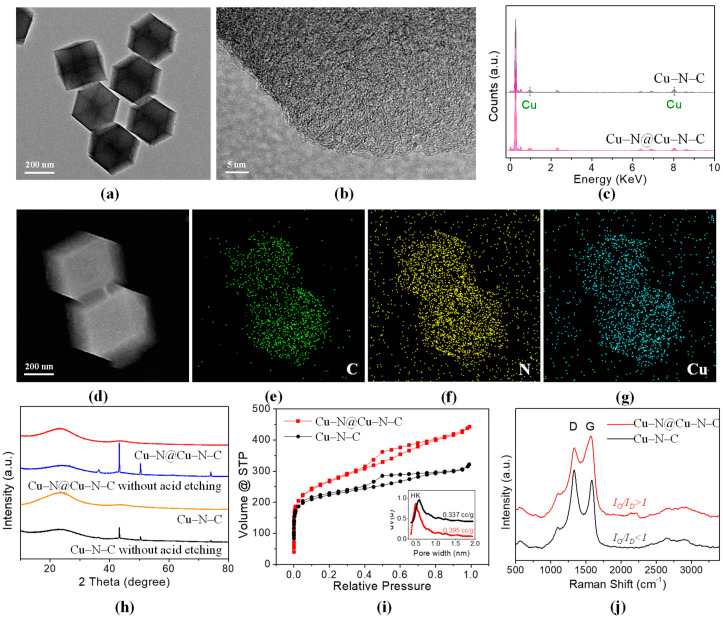
Schematic illustration showing the preparation of Cu–N–C and Cu–N@Cu–N–C catalysts. (**a**) TEM, (**b**) HRTEM, (**c**) EDS, (**d**) SEM, and elemental maps of (**e**) C, (**f**) N, and (**g**) Cu of Cu–N@Cu–N–C catalyst. (**h**) XRD patterns of Cu–N–C and Cu–N@Cu–N–C and their acid etching–free samples. (**i**) N_2_ adsorptiondesorption isotherm curves and corresponding micropore distribution of Cu–N–C and Cu–N@Cu–N–C. (**j**) Raman shift curves of Cu–N–C and Cu–N@Cu–N–C.

The characterization methods are listed in Supplementary Information Part S1. The TEM image in Figure 2a shows the typical rhombic dodecahedron structure of ZIFs, indicating the tough structural stability of Cu–N@Cu–N–C even after two-time high-temperature treatments. Furthermore, no obvious macropores or hollows were observed on the surface of Cu–N@Cu–N–C, which will be further discussed later according to the pore size distribution. The HRTEM image in Figure 2b shows that no metallic species were observed, implying the complete removal of Cu particles. In addition, only a typical graphene-like structure with a lattice distance of about 0.34 nm, which referred to the graphite (002) plane, was observed, indicating the existence of a large number of graphitic carbons in Cu–N@Cu–N–C, which was further confirmed later according to Raman shift analysis.

Energy dispersive spectroscopy (EDS) based on SEM analysis showed the existence of Cu elements in both Cu–N–C and Cu–N@Cu–N–C samples, as shown in Figure 2c. Meanwhile, the SEM image (Figure 2d) and its corresponding elemental maps (Figure 2e–g) indicated a uniform distribution of Cu and N in carbon-based nanomaterials. The uniformly elemental dispersion of transition metal and N- in ZIF-based carbon nanomaterials is usually considered as the existence of M–Nx coordinates [1,5,63,64]. The XRD patterns shown in Figure 2h indicate only two smooth peaks at about 26° and 44°, which corresponded to the (002) and (100) planes of carbon [65], respectively, in both Cu–N@Cu–N–C and Cu–N–C. However, the XRD patterns of samples without the acidic etching process showed several sharp peaks at about 43°, 50°, and 74° which corresponded to the (111), (200), and (220) planes of metallic Cu (PDF#04-0836), respectively, indicating the acidic etching process successfully removed the metallic species, which was beneficial for the possible atomic level distribution of Cu. 

The N_2_ adsorption–desorption isotherm curves of Cu–N@Cu–N–C and Cu–N–C are shown in Figure 2i, indicating a higher Brunauer–Emmett–Teller (BET) SSA of Cu–N@Cu–N–C (955.68 m^3^ g^−1^) than that of Cu–N–C (830.02 m^3^ g^−1^). The corresponding Horvath–Kawazoe (HK) micropore size distribution curves are shown in the insert chart in Figure 2i. Cu–N@Cu–N–C with a smaller micropore size (about 0.5 nm) compared with that of Cu–N–C (about 0.6 nm) was observed, preliminarily confirming that additional Cu–Nx active sites were successfully filled into the micropores of Cu–N–C, which will be further discussed later according to XPS and XAFS spectrum analysis. Moreover, the pore volume increased from 0.337 cc g^−1^ for Cu–N–C to 0.395 cc g^–1^ for Cu–N@Cu–N–C. The larger SSA, smaller micropore size, and bigger pore volume provided more space for catalytic active sites and improved the ORR performance of Cu–N@Cu–N–C compared with Cu–N–C. Raman shift curves are shown in Figure 2j. Two typical peaks were observed at 1330 cm^−1^ and 1580 cm^−1^, which corresponded to defect or disordered carbon (D) and graphitic or ordered carbon (G), respectively, in both Cu–N@Cu–N–C and Cu–N–C. However, a great difference between the two samples was found in the ratio of the intensity of the G peak to the D peak, i.e., I_G_/I_D_, which is usually related to the electron conductivity or structural stability of carbon-based materials. The results showed a much greater I_G_/I_D_ (=1.17) for Cu–N@Cu–N–C compared with that of Cu–N–C (I_G_/I_D_ = 0.84), which could be ascribed to the two-time pyrolysis for the former. This indicated the greater degree of graphitization and better electron conductivity that, to some extent, resulted in enhanced ORR performance, especially the durability of Cu–N@Cu–N–C catalyst [66,67].

### 3.2. Electrochemical Performance Tests

**Figure 3:**
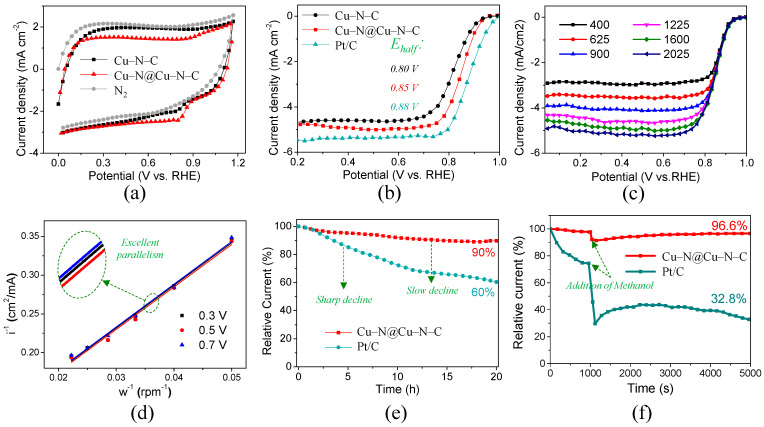
(**a**) CV and (**b**) LSV curves of Cu–N–C, Cu–N@Cu–N–C and Pt/C. (**c**) LSV curves and (**d**) corresponding K–L plots of Cu–N@Cu–N–C tested with different rotating speeds ranges from 400 to 2025 rpm. (**e**) Long-term durability and (**f**) methanol tolerance tests of Cu–N@Cu–N–C and Pt/C at 0.7 V. All the measurements are based on a 0.1 M KOH electrolyte solution.

The electrochemical measurement methods are listed in Supplementary Information Part S2. In order to evaluate whether the ORR catalytic activity of the Cu–N@Cu–N–C sample improved or not, the CV, LSV, and i-t curves were measured and compared to those of Cu–N–C and Pt/C 20 wt% in alkaline (0.1 M KOH) electrolytes. CV curves were first examined, and the results indicated peak potentials of 0.82 V and 0.78 V for Cu–N@Cu–N–C and Cu–N–C, respectively, as shown in Figure 3a. The LSV curves in Figure 3b demonstrate the on-set potentials of 0.96 V, 0.95 V, and 0.99 V for Cu–N@Cu–N–C, Cu–N–C, and Pt/C 20 wt%, respectively. The half-wave potential (E_half_) was considered the most important parameter for ORR catalytic activity. The results demonstrated E_half_ of 0.85 V, 0.80 V, and 0.88 V for Cu–N@Cu–N–C, Cu–N–C, and Pt/C 20 wt%, respectively. The great enhancement of 50 mV in E_half_ for Cu–N@Cu–N–C compared with that for Cu–N–C, verified that the origin of higher ORR performance was potentially attributed to the increase in Cu–Nx active sites. Moreover, the E_half_ of commercial Pt/C 20 wt% was only 30 mV more positive than Cu–N@Cu–N–C, confirming the excellent activity of Cu–Nx active sites. Moreover, it showed similar or better ORR activity compared with Cu species catalysts according to the literature [48,59,65]. It was noticeable that the limited current density of Cu–N@Cu–N–C was slightly higher than that of Cu–N–C, which was potentially ascribed to the higher SSA or more rational pore distribution, as discussed previously.

The ORR kinetics of the Cu–N@Cu–N–C catalyst were investigated based on LSV curves that were tested at rotating speeds, as shown in Figure 3c. The limited current density increased with the increase in rotation speeds from 400 to 2025 rpm. The Koutecky–Levich (K-L) plots shown in Figure 3d indicate excellent parallelism and linearity from the potential of 0.3 V to 0.7 V, verifying the first-order reaction kinetics that commonly represented a good ORR performance. Long-term durability was measured and shown as current–time (i-t) curves. A slow decline of 10% in current for Cu–N@Cu–N–C and a drastic decrease of 40% for Pt/C 20 wt% were observed after 20 h. This verified the superior stability of the Cu–N@Cu–N–C catalyst, which could be attributed to its high graphitization degree, as shown in Figure 3e. We investigated SEM and TEM images before and after the durability testing, as shown in Supplementary Information Figure S1 and Figure S2, respectively. No obvious changes were observed except for the smoother surface of the sample after durability testing, indicating the good structural stability of Cu–N@Cu–N–C. Methanol tolerance tests were also conducted, as shown in Figure 3. When 5 mL of 3 M methanol was dropped into the alkaline electrolyte over 1000 s, no obvious declines were found for Cu–N@Cu–N–C with a relative current of 96.6% after 5000 s, while a sharp decrease to 32.8% was observed for Pt/C 20 wt%. As shown in Supplementary Information Figure S3, methanol tolerance testing with longer exposure times was investigated, and a relative current of 94.5% after 10 000 s was observed, further confirming the excellent methanol tolerance of Cu–N@Cu–N–C.

**Figure 4:**
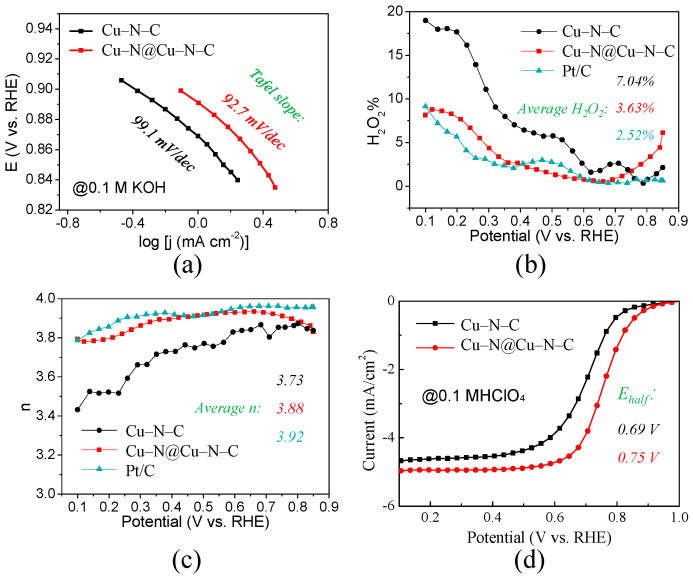
(**a**) Tafel slope curves of Cu–N–C and Cu–N@Cu–N–C based on LSV measurements in 0.1 M KOH. (**b**) Yield of H_2_O_2_ and (**c**) transferred electron number of Cu–N–C, Cu–N@Cu–N–C, and Pt/C in 0.1 M KOH. (**d**) LSV curves of Cu–N–C and Cu–N@Cu–N–C in acidic 0.1 M HClO_4_ electrolyte solution.

The Tafel plots derived from LSV curves in alkaline electrolytes are shown in Figure 4a. A slightly smaller Tafel slope was measured as 92.7 mV dec^−1^ for Cu–N@Cu–N–C compared with 99.1 mV dec^−1^ for @Cu–N–C, further demonstrating its better ORR catalytic activity. In order to evaluate the electronic transmission characteristics, i.e., four-electron transfer rate, LSV curves of the catalysts measured on the RRDE were acquired. The yield of H_2_O_2_ and the number of transferred electrons were calculated, as shown in Figure 4b and Figure 4c, respectively. The average value for the formation of H_2_O_2_ was calculated as 3.63% for Cu–N@Cu–N–C according to Formula (4) in Supplementary Information Part S2. It was much lower than calculated for Cu–N–C (7.04%) and a little higher than that calculated for Pt/C 20 wt% (2.52%). The average number of transferred electrons was calculated as 3.88 for Cu–N@Cu–N–C according to Formula (5) in Supplementary Information Part S2. It was much higher than the number calculated for Cu–N–C (transferred number 3.73) and a little lower than that calculated for Pt/C 20 wt% (transferred number 3.92). The lower yield of H_2_O_2_ and higher number of transferred electrons further confirmed the better ORR activity of Cu–N@Cu–N–C compared with Cu–N–C. Moreover, the electron transfer pathway of Cu–N@Cu–N–C in alkaline media, as described in Supplementary Information Part S3, indicates the four transfer steps. The detailed intermediate adsorption states are depicted in Supplementary Information Figure S4. 

Generally, the ORR catalytic activity in acidic media is comparable and challenging. Figure 4d shows LSV curves of the two Cu-based catalysts that were measured in 0.1 M HClO_4_ electrolyte solution. The E_half_ was tested to be 0.75 V for Cu–N@Cu–N–C and 0.69 V for Cu–N–C. Apparently, there was an obvious enhancement of 60 mV, which should be attributed to the increase in Cu–Nx active site density.

### 3.3. Chemical Structure and Active Site Analysis

**Figure 5:**
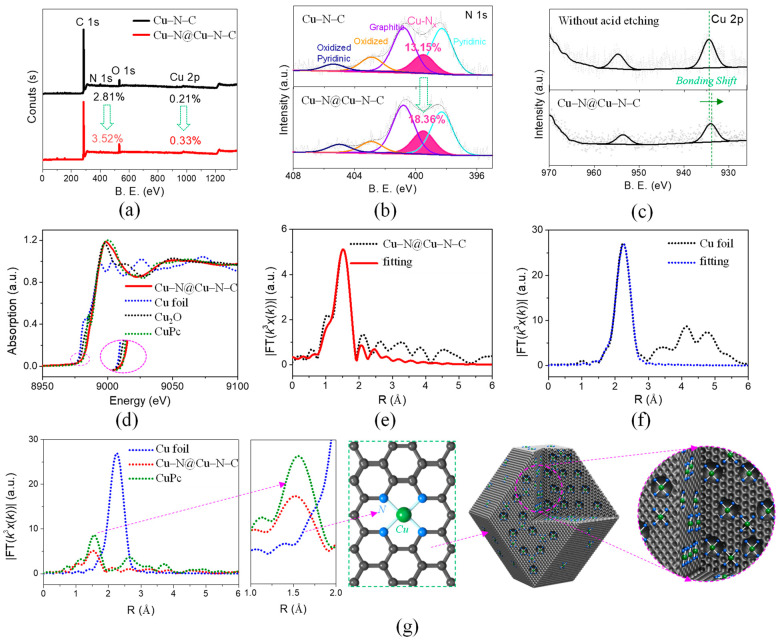
(**a**) XPS spectra evaluations of Cu–N–C and Cu–N@Cu–N–C. (**b**) High-resolution XPS patterns of N 1s spectra of Cu–N@Cu–N–C and Cu–N–C. (**c**) Cu 2p spectra of Cu–N@Cu–N–C and its counterpart sample without the acidic washing process. (**d**) XANES curves of Cu–N@Cu–N–C and its Cu foil, Cu_2_O, and Cu phthalocyanine (CuPc) counterparts. FT-EXAFS curves of the raw measurements and fitting results of (**e**) Cu–N@Cu–N–C and (**f**) Cu foil. (**g**) FT-EXAFS curves of Cu–N@Cu–N–C and its Cu foil and CuPc counterparts. The graphs beside (**g**) are the local enlarged picture of FT-EXAFS. The near pictures show the simulated chemical structure and local microscopic morphology of Cu–N@Cu–N–C.

The XPS spectrum of Cu–N@Cu–N–C and Cu–N–C are shown in Figure 5a, indicating several typical peaks at about 932 eV, 533 eV, 401 eV, and 285 eV, corresponding to Cu 2p, O 1s, N 1s, and C 1s, respectively. The rough elemental contents based on the XPS results are listed in Supplementary Information Table S1, indicating an obvious increase in the content of both N (from 2.81 at% to 3.52 at%) and Cu (from 0.21 at% to 0.33 at%) for Cu–N@Cu–N–C, compared with those for Cu–N–C. This to some extent confirmed that additional N and Cu were successfully doped into Cu–N–C nanomaterials. Furthermore, the ICP results in Supplementary Information Table S2 show a great increase in Cu content for Cu–N@Cu–N–C (1.92 wt%) compared with that for Cu–N–C (0.88 wt%), proving additional Cu was absolutely doped into the micropores of Cu–N–C. We noted a much higher value from ICP testing compared with the XPS results, which could be ascribed to the inaccurate quantitative analysis of the surface-sensitive XPS method. N species were commonly reported to significantly affect the ORR activity of M–N–C catalysts. The high-resolution N 1s spectra of the above two samples were evaluated, as shown in Figure 5b. There were five divided peaks at about 405.0 eV, 402.7 eV, 401.0 eV, 399.5 eV, and 398.4 eV, corresponding to Oxidized–Pyridinic–N (Oxidized–P–N), Oxidized–N, Graphitic–N, Cu–Nx, and Pyridinic–N, respectively. The contents of these N species are listed in Supplementary Information Table S3. An obvious increase in Cu–Nx from 13.15% (for Cu–N–C) to 18.36% (for Cu–N@Cu–N–C) was observed, proving the formation of additional Cu–Nx in the micropores of Cu–N–C. Moreover, it was noticed that the contents of Pyridinic–N decreased from 36.25% (for Cu–N–C) to 31.15% (for Cu–N@Cu–N–C), demonstrating the possible transformation of Pyridinic–N into Cu–Nx. The high-resolution Cu 2p spectra are shown in Figure 5c. A slight shift (about 0.5 eV) in the bonding energy around 934 eV was observed for Cu–N@Cu–N–C compared with its counterpart sample without the acidic washing process, which could be ascribed to the interaction between metallic clusters and Cu–Nx in the acidic washing-free sample [68]. 

To investigate the chemical coordinate state of Cu in Cu–N@Cu–N–C, XAFS was evaluated. As shown in Figure 5d, the Cu k-edge XANES results showed the pre-edge position of Cu–N@Cu–N–C was far more positive than its Cu foil (Cu with 0 valence state) and Cu_2_O (Cu with +1 valence state) counterparts and was very close to Cu phthalocyanine (CuPc, Cu with +2 valence state), implying the chemical valence state of Cu in Cu–N@Cu–N–C was near +2. The Fourier-transformed k3-weighted extended X-ray absorption fine structure (FT-EXAFS) of Cu–N@Cu–N–C is shown in Figure 5e. The fitting result indicated only a typical peak at about 1.5 Å, which confirmed the single-atom dispersion of Cu. The FT-EXAFS of Cu foil and its fitting result revealed a dominant peak at about 2.2 Å, as shown in Figure 5f. The FT-EXAFS fitting results of Cu–N@Cu–N–C and its Cu foil and CuPc counterparts showed exactly the same typical peak position at about 1.5 Å between Cu–N@Cu–N–C and CuPc, implying the similar chemical coordinate states for them, i.e., Cu was coordinated with N atoms to form Cu–Nx species, as shown in Figure 5g and the two graphs to its right. Furthermore, the fitting results based on EXAFS analysis showed that Cu was coordinated by 3.6 (±0.2) N atoms with the bonding distance of 1.95 (±0.01) Å, confirming the predominant existence of Cu–N_4_ species that were reported to act as outstanding active centers in ORR, as shown in Supplementary Information Table S4. Moreover, the high-density Cu–N_4_ active sites fastened the rate-determining step (i.e., the formation of OOH) of ORR for our Cu–N@Cu–N–C catalyst. Combined with the above discussion, the simulated distribution of Cu–N_4_ on the surface as well as in the micropores of Cu–N@Cu–N–C was obtained, as shown in the two graphs on the far right in Figure 5g. It could be concluded that the improvement in ORR performance of Cu–N@Cu–N–C compared with Cu-N-C was attributed to the enlargement in Cu–N_4_ active site density.

## 4. Conclusions

An efficient single-atom Cu–N@Cu–N–C catalyst was developed by a second doping and pyrolysis method, which significantly enlarged the Cu–Nx active site density based on Cu–N–C. The filling of Cu salts and organic ligands into micropores of Cu-N-C was evaluated using BET and HK micropore analysis. The second doping of Cu and formation of additional Cu–Nx were confirmed by the ICP and XPS results, respectively. The atomically dispersed Cu–N_4_ catalytic centers were verified by XAFS analysis. With the enriched Cu–N_4_ active centers, ORR performance experiments with Cu–N@Cu–N–C indicated more positive E_half_ in both alkaline and acidic media, a lower yield of H_2_O_2,_ and higher four-electron selectivity, compared with those of Cu–N–C. Particularly, this as-prepared Cu–N@Cu–N–C showed the desired first-order reaction kinetics, superior long-term durability, and resistance to methanol toxicity compared with the commercial Pt-based catalyst in alkaline electrolytes. The enhanced catalytic activity of Cu–N@Cu–N–C was believed to be attributed to the enlargement in atomically dispersed Cu–N_4_ active site density in three-dimensional carbon nanostructures. This study provides an effective route to develop practical Pt-free ORR catalysts with high M–N_x_ active site density, which is beneficial for their potential application in hydrogen energy fuel cells or metal–air batteries.

## Data Availability

Data will be made available upon request.

## Supplementary Materials

The following supporting information can be downloaded at: https://www.mdpi.com/article/10.3390/ma17205030/s1, Part S1: Materials characterization; Part S2: Electrochemical measurements; Part S3: Electron transfer pathways; Figure S1: SEM images of Cu-N@Cu-N-C before and after durability testing; Figure S2: TEM images of Cu-N@Cu-N-C after durability testing; Figure S3: Methanol tolerance testing for Cu-N@Cu-N-C; Figure S4: Schematic diagram on electron transfer pathways of Cu-N@Cu-N-C; Table S1: Elemental contents of Cu–N@Cu–N–C and Cu–N–C based on XPS results; Table S2: ICP results of Cu–N@Cu–N–C and Cu–N–C; Table S3: N types and contents of Cu–N@Cu–N–C and Cu–N–C based on N 1s results; Table S4: EXAFS data fitting results of Cu–N@Cu–N–C and Cu foil.
