# Supplementary material for: Atomically Dispersed High-Active Site Density Copper Electrocatalyst for the Reduction of Oxygen
Source: Materials (Basel). 2024 Oct 15;17(20):5030. doi: 10.3390/ma17205030 (PMC11509251; doi:10.3390/ma17205030)
Supplement: Supplementary file 1 [file materials-17-05030-s001.zip › materials-3221835-supplementary.pdf]

Supplementary Information

Atomically Dispersed High-Active Site Density Copper Electrocatalyst for the Reduction of Oxygen

Part S1. Material characterization

Scanning electron microscopy (SEM, Hitachi Limited S–4800), transmission electron microscopy (TEM, JEOL JEM–2100F), high-angle annular dark field scanning transmission electron microscopy (HAADF–STEM, Hitachi S–5500), and aberration-corrected transmission electron microscopy (ACTEM, FEI Titan Themis TEM) were used to investigate the morphology of the samples. Powder X-ray diffraction (XRD, Rigaku 2550VB) was used to analyze the crystal structure of the samples. Raman spectra were recorded on an Invia/Reflrx Laser spectrometer (Renishaw, England) excited by a laser beam of 532 nm. Inductively coupled plasma mass spectrometry (ICP MS, Baird PS–6) was used to measure the metal element content of the samples. The nitrogen adsorption and desorption method (Mike ASAP 2020 HD88) was used to investigate the specific surface area and pore size distribution. X-ray photoelectron spectroscopy (XPS) with an Al Kα X-ray source (Thermo ESCALAB250) was used to determine the chemical state and valence of the samples. X-ray adsorption fine spectroscopy (XAFS) was used to investigate the fine chemical environment of the samples. Extended X-ray absorption fine structure (EXAFS) was carried out at the Catalysis and Surface Science Endstation at the XAFCA beamline in the Synchrotron Light Source (SSLS) in Singapore. The EXAFS spectra were recorded using the Gaussian window function. The Cu K-edge X-ray near-edge structure (XANES) data were recorded in fluorescence mode. The detector was an ion chamber with a double-crystal Si (111) monochromator. The storage ring operated at the energy of 700 MeV with an average electron current of 200 mA.

Part S2. Electrochemical measurements

All electrochemical measurements in this article are based on the typical three-electrode system with a rotating disk electrode (RDE, 5 mm in diameter) or a rotating ring-disk electrode (RRDE, 4 mm in diameter for the disk and 1 mm for the ring) as the working electrode, Ag/AgCl (over-saturated KCl) as the reference electrode, and graphite rod as the counter electrode. All the potentials in our manuscript were vs. RHE. The conversion formula was E (vs. RHE) = E (vs. Ag/AgCl) +0.197 V+0.0591×pH. The pH of our alkaline and acidic electrolytes was 13.1 and 1.3, respectively. The calculations were as follows:

0.1 M KOH: E (vs. RHE) = E (vs. Ag/AgCl) + 0.97 V.

0.1 M HClO4: E (vs. RHE) = E (vs. Ag/AgCl) + 0.27 V.

Pt-free catalyst inks were prepared by dissolving 10 mg of Cu–N@Cu–N–C or Cu–N–C into a mixed solution containing 60 μL of Nafion, 470 μL water, and 470 μL ethanol. The Pt/C catalyst ink was prepared by dispersing 5 mg Pt/C 20wt% into 960 μL of isopropanol and 40 μL of Nafion. The working electrode was prepared by dropping 5 μL of Cu–N@Cu–N–C or Cu–N–C onto the RRDE or 8 μL onto the RDE to reach a loading of 0.4 mg cm^{-2}. The electrolytes for acid and alkaline media were 0.1 M $HClO_4$ and 0.1 M KOH, respectively. All

the tests were based on an O_2-saturated solution. For the cyclic voltammetry (CV) test, the potential range was set from –1 V to 0.2 V at a sweep rate of 50 mV s^{-1} in alkaline media (–0.2 to 1 V in acidic media). Linear sweep voltammetry (LSV) curves were recorded on the RRDE or RDE (rotating rates of 1600 rpm) from 0.2 V to –1 V with a sweep rate of 10 mV s^{-1} for alkaline media (1 to –0.2 V for acidic media). The electron transfer number, *n*, was calculated using Koutecky–Levich equations as follows:

$$\frac{1}{j} = \frac{1}{j_L} + \frac{1}{j_K} = \frac{1}{Bw^{1/2}} + \frac{1}{j_K} \quad (1)$$

$$B = 0.2nFC_0(D_0)^{2/3}v^{-1/6} \quad (2)$$

$$j_K = nFkC_0 \quad (3)$$

where j, j_L, and j_K are the measured diffusion-limiting and kinetic current densities, respectively, ω is the rotating rate of the RRDE or RDE (rpm), C_0 is the bulk concentration of O_2, F is the Faraday constant, D_0 is the diffusion coefficient of O_2, and v is the kinematic viscosity of the 0.1 M KOH solution. The yield of H_2O_2 and the value of n were calculated using the following equations:

$$\%H_2O_2 = \frac{200\frac{I_{ring}}{N}}{\frac{I_{ring}}{N}+I_{disk}} \quad (4)$$

$$n = \frac{4I_{disk}}{\frac{I_{ring}}{N}+I_{disk}} \quad (5)$$

where I_{disk} and I_{ring} are the current density of the disk and ring of the RRDE, respectively, and N is the collection efficiency (0.39) of the ring electrode measured in 1 M potassium ferricyanide ($K_3Fe(CN)_6$) and 0.1 M KCl solutions. Note that the ring electrode potential was set at 0.51 V (vs. Ag/AgCl).

Part S3. Electron transfer pathways

In the alkaline medium, the ORR of Cu-N@Cu-N-C may involve the following four-electron steps:

$O_2 + H_2O + e^- + * \rightarrow OOH^* + OH^-$ (Step 1).

$OOH^* + e^- \rightarrow O^* + OH^-$ (Step 2).

$O^* + H_2O + e– \rightarrow OH^* + OH^-$ (Step 3).

$OH^* + e^- \rightarrow * + OH^-$ (Step 4).

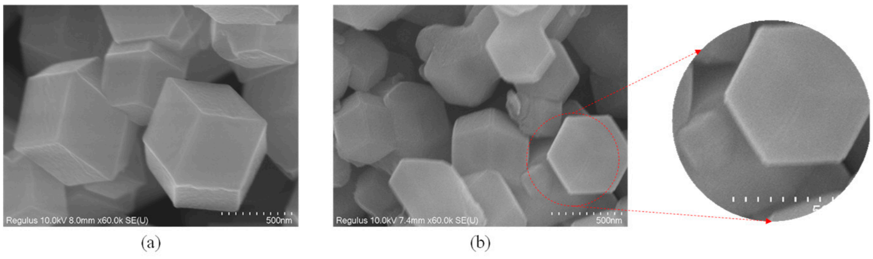

Figure S1. SEM images of Cu-N@Cu-N-C (a) before and (b) after durability testing.

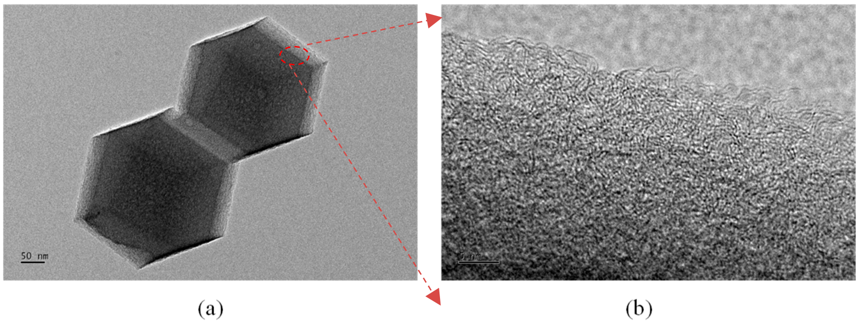

(a) (b)

Figure S2. TEM images of Cu-N@Cu-N-C after durability testing.

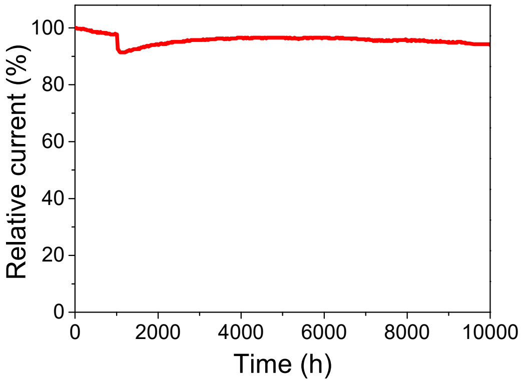

Figure S3. Methanol tolerance testing for Cu-N@Cu-N-C.

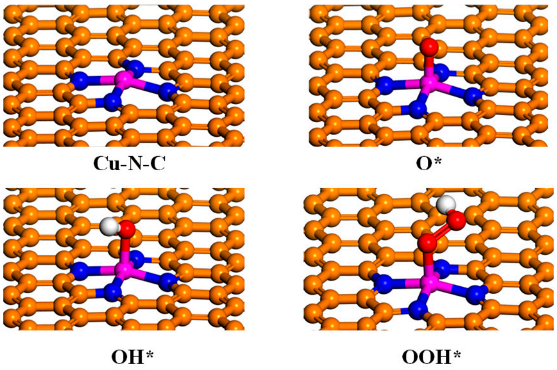

Figure S4. Schematic diagram of the electron transfer pathways of Cu-N@Cu-N-C.

Table S1. Elemental contents of Cu–N@Cu–N–C and Cu–N–C based on XPS results.

Sample	C	N	O	Cu
Cu–N@Cu–N–C	90.18 at%	3.52 at%	5.97 at%	0.33 at%
Cu–N–C	89.27 at%	2.81 at%	7.71 at%	0.21 at%

Table S2. ICP results of Cu–N@Cu–N–C and Cu–N–C.

Sample	Mass	Volume	Element	Content
Cu–N@Cu–N–C	67 mg	100 mL	Cu	1.92 wt%
Cu–N–C	62 mg	100 mL	Cu	0.88 wt%

Table S3. N types and contents of Cu–N@Cu–N–C and Cu–N–C based on N 1s results.

Sample	Pyridinic–N	Cu–Nx	Graphitic–N	Oxidized–N	Oxidized–P–N
Cu–N@Cu–N–C	31.15 %	18.36 %	36.28 %	8.26 %	5.95 %
Cu–N–C	36.25 %	13.15 %	34.46 %	9.97 %	6.17 %

Table S4. EXAFS data fitting results of Cu–N@Cu–N–C and Cu foil.

Sample	shell	*CN*	*R* (Å)	σ^2 (Å^2)	ΔE_0 (eV)	R factor
Cu–N@Cu–N–C	Cu–N	3.6±0.2	1.95±0.01	0.0063	3.0±1.6	0.0090
Cu foil	Cu–Cu	12	2.54±0.01	0.0087	5.4 ±0.5	0.0028
